# Endothelin-2/Vasoactive Intestinal Contractor: Regulation of Expression via Reactive Oxygen Species Induced by CoCl_2_2, and Biological Activities Including Neurite Outgrowth in PC12 Cells

**DOI:** 10.1100/tsw.2006.37

**Published:** 2006-02-17

**Authors:** Eiichi Kotake-Nara, Kaname Saida

**Affiliations:** ^1^ Institute for Biological Resources and Functions National Institute of Advanced Industrial Science and Technology (AIST), Tsukuba Central 6, 1-1-1 Higashi, Ibaraki 305-8566, Japan; ^2^ New Energy and Industrial Technology Development Organization (NEDO), 16F, MUZA KAWASAKI Central Tower, 1310 Omiya-cho, Saiwai-ku, Kawasaki, Kanagawa 212-8554, Japan

**Keywords:** cobalt chloride (CoCl_2_), endothelin-1 (ET-1), endothelin-2 (ET-2), hypoxia, neurite outgrowth, PC12, reactive oxygen species (ROS), vasoactive intestinal contractor (VIC)

## Abstract

This paper reviews the local hormone endothelin-2 (ET-2), or vasoactive intestinal contractor (VIC), a member of the vasoconstrictor ET peptide family, where ET-2 is the human orthologous peptide of the murine VIC. While ET-2/VIC gene expression has been observed in some normal tissues, ET-2 recently has been reported to act as a tumor marker and as a hypoxia-induced autocrine survival factor in tumor cells. A recently published study reported that the hypoxic mimetic agent CoCl_2_ at 200 µM increased expression of the ET-2/VIC gene, decreased expression of the ET-1 gene, and induced intracellular reactive oxygen species (ROS) increase and neurite outgrowth in neuronal model PC12 cells. The ROS was generated by addition of CoCl_2_ to the culture medium, and the CoCl_2_-induced effects were completely inhibited by the antioxidant *N*-acetyl cysteine. Furthermore, interleukin-6 (IL-6) gene expression was up-regulated upon the differentiation induced by CoCl2. These results suggest that expression of ET-2/VIC and ET-1 mediated by CoCl_2_-induced ROS may be associated with neuronal differentiation through the regulation of IL-6 expression. CoCl_2_ acts as a pro-oxidant, as do Fe(II, III) and Cu(II). However, some biological activities have been reported for CoCl_2_ that have not been observed for other metal salts such as FeCl_3_, CuSO_4_, and NiCl_2_. The characteristic actions of CoCl_2_ may be associated with the differentiation of PC12 cells. Further elucidation of the mechanism of neurite outgrowth and regulation of ET-2/VIC expression by CoCl_2_ may lead to the development of treatments for neuronal disorders.

[*]Corresponding author.
©2006 with author.
Published by TheScientificWorld, Ltd.; www.thescientificworld.com

## INTRODUCTION

Endothelin-1 (ET-1), a vasoconstrictor peptide produced by vascular endothelial cells[1], endothelin-2 (ET-2), endothelin-3 (ET-3), and vasoactive intestinal contractor (VIC) are members of the ET family. These peptides each contain two characteristic disulfide bridges (Fig. 1). VIC is thought to be the mouse and rat orthologous peptide of the human ET-2[2,3,4]. In many tissues, expression of ET-1 is predominant among the ET peptides[5,6,7,8]; therefore, most studies of ET peptides have focused on ET-1. ET-2/VIC gene expression, however, has been observed at high levels in the intestine, ovary, uterus, stomach, testis, and cerebrum[5,6,7,8]. Thus, it is expected that ET-2/VIC has some important physiological functions in these organs. This review describes the distribution and expression of ET-2/VIC in biological systems.

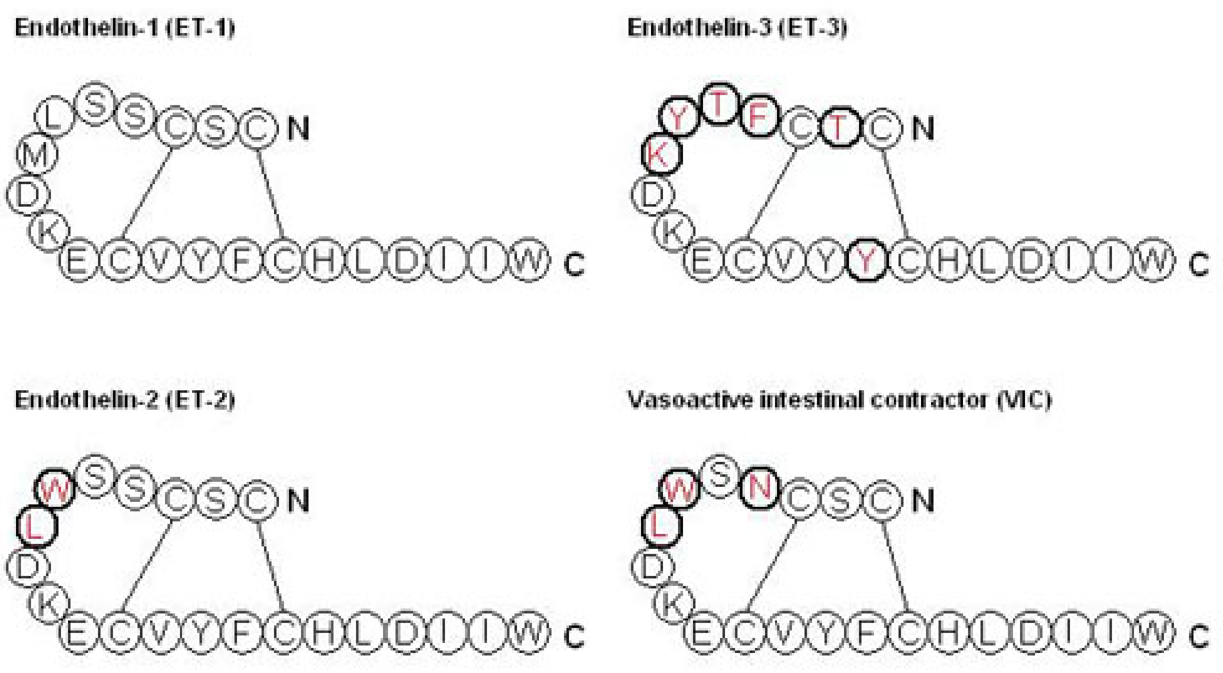

**FIGURE 1.** Structures of ET family peptides. Lines indicate disulfide bridges. Bold circles with red font indicate differences from ET-1 among amino acid residues. The peptide sequences are derived from mouse and rat (ET-1, VIC, and ET-3) or human (ET-1, ET-2, and ET-3) species, respectively.

Recently ET-2 has been reported to act as a cancer-related[9,10,11,12] and as a hypoxia-related[12,13] factor. Hypoxia stimulates the invasiveness of[14] and up-regulates telomerase activity in[15] cancer cells. Moreover, it is thought that hypoxia attenuates the generation of reactive oxygen species (ROS) that is caused by irradiation or chemical agents, thereby reducing the effectiveness of these interventions in the treatment of cancer. Although the expression of ET-2/VIC as a survival factor induced by hypoxia may have negative effects on the results of cancer treatment, it may act in a positive way against neuronal diseases caused by cell death. PC12, a rat pheochromocytoma cell line, is widely used as a model for neuronal differentiation. This review, in addition to examining the functions of ET-2/VIC, also describes the relationship between the ET-2/VIC up-regulation and the cell growth, neurite outgrowth, cell death, and ROS generation induced by cobalt chloride ($CoCl_2$), a well known hypoxia mimetic agent[16], in a PC12 neuronal model.

## DISCOVERY, DISTRIBUTION, AND EXPRESSION OF ET-2 AND VIC

ET-2 and VIC were discovered independently in the late 1980s through analysis of human[2] and mouse[3] genomes. The structure of ET-2 differs from that of VIC by only 1 of 21 amino acid residues (Fig. 1). Southern blot analysis, cloning, and sequencing indicate that VIC is the mouse and rat orthologous peptide of the human ET-2[2,3,4]. ET-1 is known to be produced in a wide variety of biological systems. VIC, on the other hand, based on Northern blot analysis of mouse VIC gene expression[3], was at first known to exist only in the intestine. Subsequently, more sensitive RT-PCR and real-time PCR analyses[5,6,7,8] revealed that ET-2/VIC is expressed not only in the intestine, but also in the ovary, uterus, stomach, testis, and cerebrum. Furthermore, the VIC gene has been shown to be expressed at high levels in the pituitary gland of the murine central nervous system, although the VIC expression level is lower than that of the ET-1 gene[17].

In addition to PCR gene expression analysis, peptide levels of ET family ligands by antibody or reverse-phase HPLC[18,19] have been reported. A commercial kit that employs a sandwich ELISA measurement of ET-1 peptide production is currently available. However, not only is the antibody provided with the kit unable to distinguish between ET-1 and ET-2[20], but it also has cross-reactivity with ET-3. Thus, antibody analysis alone is unable to quantitatively determine distinct levels of ET-1 and ET-2. However, we recently reported the use of a highly specific ET-2/VIC antibody that does not bind ET-1[21,22] and found, through immunostaining, that ET-2/VIC peptide is produced in mouse skin[23] and intestine[22]. The development of techniques for detecting ET-2/VIC peptide based on this new antibody deserves further study.

## BIOLOGICAL ACTIONS OF ET-2/VIC

The biosynthetic pathway for VIC was proposed based on DNA cloning analysis[6]. Mature VIC of 21 amino acids is produced via big VIC through the processing of prepro (PP) VIC (Fig. 2). It is well known that ET receptors, type A (ET-A) or type B (ET-B), mediate ET function. ET-B binds the three peptides, ET-1, ET-2, and ET-3, with almost equal affinities, while ET-A binds ET-1 most strongly, followed by ET-2 and then ET-3[24,25]. There is little information, however, concerning the affinities of VIC against these two receptors. VIC was reported to bind to a receptor present in Swiss mouse 3T3 cells with the same affinity as ET-1, although the receptor was not identified as either ET-A or ET-B[26,27]. The receptor was thought to be ET-A, however, because the affinity of ET-3 for the receptor was much less than that of ET-1 or VIC[27]. These reports suggest that VIC binds to ET-A with the same affinity as ET-1. Determination of the affinity of VIC against both ET receptors deserve future study.

Mice with ET system deficiencies have been developed and reported. ET-1 homozygous knockout mice die at birth and possess craniofacial abnormalities such as deformed thyroid cartilage, missing hyoid and a large part of the tongue, and cleft palate[28], as well as malformations of the aortic arch and septal ventricular defect[29]. Elevated blood pressure was observed in ET-1 heterozygous mice[28]. Mice deficient in ET-A also showed craniofacial deformities and defects in the cardiovascular outflow tract caused, respectively, by disruption of cephalic and cardiac neural crest development[30]. Mice deficient in ET-3[31] or ET-B[32] showed a distension of the intestine (megacolon) and coat color spotting related to deficient development of enteric ganglion neurons and epidermal and choroidal melanocytes, respectively. These results indicate that ET peptides and receptors are essential components in the development of normal tissue and ontogeny. The generation of an ET-2/VIC knockout mouse has not yet been reported. Thus, the essential physiological functions of ET-2/VIC remain unknown, although a number of biological actions of ET-2/VIC have been reported.

The relative vasoconstrictor activities for ETs (in anesthetized rats *in vivo*) were reported to be ET-2 > ET-1 > ET-3[2]. ET-2/VIC also exhibits other biological activities such as the induction of contraction in mouse ileum[33] and acetylcholine release in guinea-pig ileum[34,35], the stimulation of an increase in intracellular $Ca^{2+}$ in Swiss 3T3 cells[27,34] and in mouse NG108-15 neuroblastoma cells[36,37], the accumulation of diacylglycerol in NG108-15 cells and vascular smooth-muscle cells[37], and the production of both vasodilation and vasoconstriction in the systemic vascular bed and biphasic changes in pulmonary vascular resistance in the cat[38]. ET-2/VIC gene expression also increases significantly during embryonic development[5,6], suggesting that ET-2/VIC plays an important role at birth.

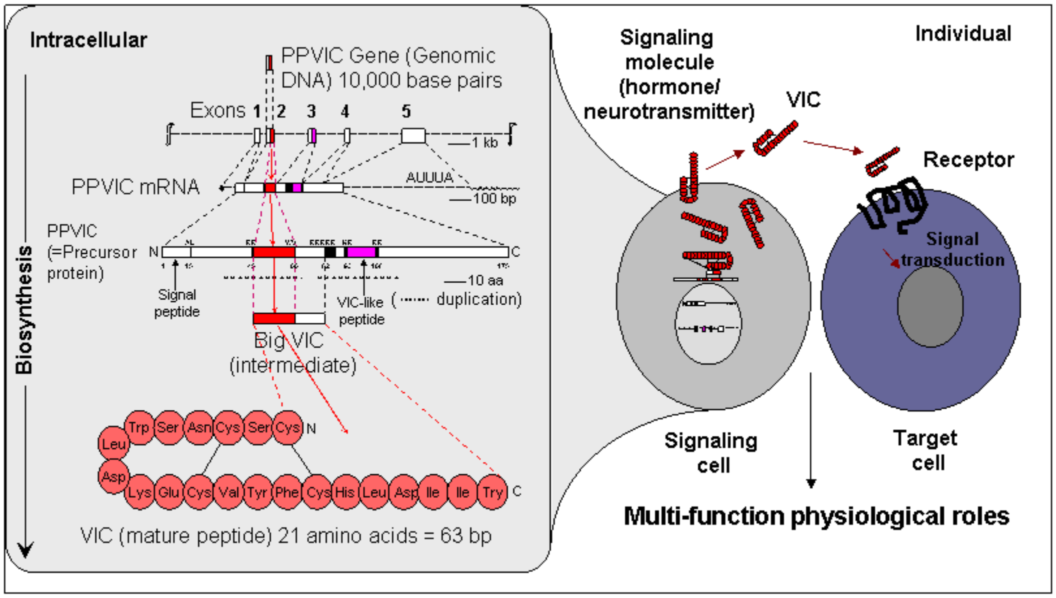

**FIGURE 2.** Biosynthetic pathway and biosignaling system of ET-2/VIC. Physiologically active mature peptide VIC/ET-2 is processed from the precursor protein, prepro ET-2/VIC, in 2 steps: from prepro-form to big intermediate form by an endopeptidase and from big form to mature peptide by a converting enzyme. The VIC/ET-2 gene, composed of 5 exons, is localized on chromosome 4 in mouse[74], chromosome 5 in rat[75], and chromosome 1 in human[4,76]. A nucleotide region for the mature peptide is located in the second exon. Alternative splicings are reported[77,78]. Multifunction physiological roles such as embryonic development and reproduction, in addition to its effects on the gastrointestinal system, are exhibited via receptors in a paracrine (right side), autocrine, or intracrine manner.

Recently ET-2 was reported to act as a tumor marker in uveal melanoma cells[9]. This marker is one of the genes most highly up-regulated by hypoxia among 588 genes tested in two different squamous carcinoma cell lines[13]. ET-2 also acts as a macrophage chemoattractant[10], an inducer of tumor cell invasion[11], and a hypoxia-induced autocrine survival factor in human breast tumor cells[12].

Hypoxia is generally thought to diminish the effects of treatments against tumors such as irradiation and chemical therapy. Moreover, hypoxia is reported to stimulate the invasiveness of MDA-MB-231 breast carcinoma cells[14] and to up-regulate telomerase activity in A2780 human ovary cancer cells and HT-29 human colorectal adenocarcinoma cells[15]. Therefore, the expression of ET-2/VIC as a survival factor induced by hypoxia is thought to have negative effects on cancer treatment. However, ET-2/VIC may be a beneficial factor in diseases caused by cell death.

## NEURITE OUTGROWTH AND REGULATION OF ET-2/VIC INDUCED BY $COCL_2$ IN PC12 CELLS[21]

Neurite outgrowth in rat cerebellar macroneurons is enhanced by ethanol[39], which acts as a hypoxia-inducible factor-1α (HIF-1α) activator[40]. Thus, hypoxia or hypoxia-inducible agents may induce neurite outgrowth in neuronal cells. Moreover, it has been suggested that neuronal differentiation of P19 mouse embryonal carcinoma cells is associated with the ET system[41]. However, it is unknown whether ET-2/VIC, one of the hypoxia-related genes[13], participates in neuronal differentiation in these cases. PC12, a rat pheochromocytoma cell line, is widely used as a model for neuronal differentiation, neuronal apoptosis, and oxygen sensor mechanisms. This cell line also has been used to screen compounds with the potential to induce neuronal differentiation. ET-2/VIC and ET-1 gene expression induced by cobalt chloride ($CoCl_2$), a well known hypoxia mimetic agent, in PC12 cells has been studied. The association of ET-2/VIC and ET-1 gene expression with cell growth, neurite outgrowth, or PC12 cell death induced by $CoCl_2$ also was evaluated. On the basis of morphological observations and immunofluorescence staining with neurofilament antibody, it was shown that 200 μ*M* $CoCl_2$ inhibited cell viability and induced neurite outgrowth in PC12 cells[21]. This finding suggests that neuronal differentiation causes the inhibition of cell growth induced by $CoCl_2$ at 200 μ*M*, where cell growth was evaluated by MTT assay[42]. Furthermore, RT-PCR analysis showed that 200 μ*M* $CoCl_2$ increased ET-2/VIC gene expression in PC12 cells, while at the same time decreasing ET-1 gene expression. Immunostaining with an ET-2/VIC-specific antibody also revealed that 200 μ*M* $CoCl_2$ up-regulated ET-2/VIC peptide production. Thus, the expression of ET-2/VIC may support neuronal differentiation, despite the fact that expression of ET-2/VIC as a survival factor is thought to have an adverse effect on cancer treatment[11,12].

Up-regulation of ET-1 gene expression has been demonstrated in primary rat cardiomyocytes treated with $CoCl_2$ at 100 μ*M*[43] and in lung tissue of mice exposed to hypoxia[44], whereas up-regulation of the ET-1 gene in PC12 cells treated with $CoCl_2$ was not observed[21]. Response of the ET-1 gene to hypoxia in the cardiopulmonary system may be different from that in the PC12 neuronal model.

$CoCl_2$ at concentrations higher than 300 μ*M* significantly reduced the viability of PC12 cells[21]. Morphological observations by fluorescence microscopy with Hoechst 33342 and propidium iodide indicated that $CoCl_2$ at 500 μ*M* induced necrosis, but not apoptosis, in PC12 cells. DNA extracted from PC12 cells treated with $CoCl_2$ at concentrations higher than 300 μ*M* for 72 h revealed no apoptotic DNA ladder. These observations suggest that biological actions induced by treatment with $CoCl_2$ depend on the concentration of $CoCl_2$ in the culture medium. RT-PCR analysis produced no ET-2/VIC band in PC12 cells exposed to 500 μ*M* $CoCl_2$ for 24 h. ET-2 has been shown to be down-regulated in the apoptosis induced by ultraviolet B irradiation in the human keratinocyte[45]. Down-regulation of ET-2/VIC expression and up-regulation of ET-1 expression also was reported in mouse brain with cold injury[17]. ET-2/VIC may be associated with the inhibition of cell death induction in these types of cells or tissues. On the contrary, ET-2/VIC gene expression increases while ET-1 gene expression decreases in the differentiation of PC12 cells. These observations suggest that the regulation of ET-2/VIC and ET-1 expression might be inverse effects.

Not only nerve growth factor (NGF), but also IL-6, have been known to induce neuronal differentiation in PC12[46]. Irradiation was reported to induce neuronal differentiation in PC12 cells through the regulation of IL-6[47]. Moreover, IL-6 also was reported to protect PC12 against cell death[48]. $CoCl_2$ at 200 μ*M* up-regulates IL-6 gene expression in the differentiation of PC12 cells. Furthermore, although acetylcholinesterase activity was reported to increase in the differentiation of PC12 cells induced by NGF[49], $CoCl_2$ failed to increase acetylcholinesterase activity[50] (unpublished data). These results are consistent with the observation of differentiation induced by IL-6[46], suggesting that IL-6 is responsible for the neuronal differentiation of PC12 cells induced by $CoCl_2$. In the differentiation induced by $CoCl_2$ at 200 μ*M*, ET-2/VIC gene expression was up-regulated at an early stage, followed by the induction of IL-6 gene expression at a later stage, suggesting that ET-2/VIC might induce IL-6 expression. Treatment with 500 μ*M* $CoCl_2$ for 24 h down-regulated IL-6 as well as ET-2/VIC gene expression in PC12 cells. Similar results were reported in an earlier study: both IL-6 and ET-2 genes were shown to be down-regulated on apoptosis induction[45]. The relationship between ET-2/VIC and IL-6 remains to be elucidated and warrants future study.

ET-2/VIC was suggested to be an important factor in the regulation of both neuronal differentiation and neuronal cell death in the nervous system. ET-A and ET-B genes were expressed in PC12 cells. Which of these receptors mediates the induction of neuronal differentiation or neuronal cell death through the regulation of ET-2/VIC and ET-1 in PC12 cells is not yet known. The detailed mechanisms of mediation through ET receptors deserve further study.

Further elucidation of the mechanisms of neurite outgrowth and regulation of ET-2/VIC expression induced by $CoCl_2$ may lead to the development of treatments against neuronal disorders such as Alzheimer's and Parkinson's diseases.

## REACTIVE OXYGEN SPECIES (ROS) GENERATION BY $COCL_2$ IN THE DIFFERENTIATION OF PC12 CELLS[21]

It has been reported that intracellular ROS are responsible for the neuronal differentiation of PC12 cells induced by NGF[51]. Hypoxia also has been shown to increase the level of intracellular ROS[52,53]. Treatment with $CoCl_2$ at a concentration higher than 100 μ*M* for 1 h was reported to increase the level of ROS in PC12 cells[54]. However, it also has been shown that treatment with $CoCl_2$ at 100 μ*M* for 3 h or 20 h does not alter or decrease, respectively, the level of intracellular ROS in PC12 cells[55]. These reports suggest that exposure of PC12 cells to $CoCl_2$ may produce intracellular ROS at an early incubation time of less than 3 h. In fact, intracellular ROS, detected with the fluorescence probe diacetoxymethyl 6-carboxy-2',7'-dichlorodihydrofluorescein diacetate (DCDDF-DA), were increased in PC12 cells through treatment with low doses of $CoCl_2$ early during the incubation period, whereas the ROS level was not significantly changed by $CoCl_2$ treatments at concentrations greater than 200 μ*M*. Moreover, the differentiation induced by 200 μ*M* $CoCl_2$ was inhibited by 20 m*M* of the antioxidant NAC, and NAC also inhibited both up-regulation of the ET-2/VIC gene and down-regulation of the ET-1 gene in PC12 cells treated with $CoCl_2$ at 200 μ*M*. These results strongly suggest that the regulation of ET-2/VIC and ET-1 genes is a factor in the differentiation induced by $CoCl_2$ in PC12 cells and that regulation of ET-2/VIC and ET-1 genes is mediated by ROS in PC12 cells treated with $CoCl_2$. On the other hand, the necrosis of PC12 cells induced by $CoCl_2$ might be independent of the increase of intracellular ROS, because the ROS level in PC12 cells treated with 500 μ*M* $CoCl_2$ was comparable to that in cells treated with 200 μ*M* $CoCl_2$, and 20 m*M* NAC did not at all inhibit the reduction of cell viability or the morphological change caused by treatment with $CoCl_2$ at 500 μ*M*.

ROS derived from NADPH oxidase have been reported to be involved in NGF-induced differentiation in PC12 cells[51], and an increase in intracellular ROS caused by hypoxia also was reported to be derived from mitochondria or NADPH oxidase[52]. Involvement of ROS from mitochondria or NADPH oxidase in the differentiation induced by $CoCl_2$ has been studied (unpublished data). $CoCl_2$ did not induce the loss of mitochondrial membrane potential, which was evaluated using rhodamine 123 as a probe, indicating that ROS from mitochondria were not involved in differentiation. On the other hand, the participation of NADPH oxidase in differentiation could not be confirmed, because diphenyleneiodonium chloride (DPI), an NADPH oxidase inhibitor, exhibited strong cytotoxicity against PC12 cells. At a concentration between 0.01 and 20 μ*M*, at least, DPI did not inhibit differentiation. Recently, DPI was reported to induce apoptosis in HL-60 cells[56]. DPI might reduce cell viability through apoptosis in PC12 cells under the conditions tested. Lipid peroxidation induced by the pro-oxidant action of $CoCl_2$[57] also was thought to be one of possible sources of the ROS generation. However, ROS was generated at a very early stage. ROS was rapidly generated by the addition of $CoCl_2$ to the culture medium alone, indicating that the $CoCl_2$-triggered increase in intracellular ROS derives from ROS generated by an interaction between $CoCl_2$ and the culture medium itself. Catalase and superoxide dismutase (SOD) failed to decompose ROS generated by $CoCl_2$ in the culture medium (unpublished data), indicating that the ROS was neither hydrogen peroxide nor superoxide. Thus, the true character of the ROS in $CoCl_2$-induced differentiation in PC12 cells remains unclear and deserves further study. Even though the mechanism for the modulation of ET-2/VIC expression by ROS remains unknown, a link between ET-2/VIC gene expression and ROS generation has been shown for the first time[21].

The relationship between ET-1 regulation and ROS generation in cultured cells has been reported in several studies. Expression of ET-1 gene or peptide decreases with the addition of $H_2O_2$ to the medium or with the enhancement of intracellular ROS levels in bovine carotid artery endothelial cells[58], bovine pulmonary artery endothelial cells[59], and primary rabbit endothelial cells[60]. For PC12 cells treated with 200 μ*M* $CoCl_2$, ET-1 expression decreases with an increase in intracellular $H_2O_2$. However, ET-1 expression also has been reported to increase with $H_2O_2$ in human umbilical vein endothelial cells[61,62] and primary neonatal rat cardiac fibroblasts[63]. Thus, the mechanistic details of the relationship between ET-1 expression and ROS generation remain unclear.

## COMPARISON OF THE BIOLOGICAL ACTIONS OF $COCL_2$ WITH THOSE OF OTHER METAL SALTS

The differentiation effects of various divalent metal salts on PC12 cells have been studied extensively[64]. Whereas $MnCl_2$ induces neurite outgrowth in PC12 cells, $MgCl_2$, $CdCl_2$, $CuSO_4$, $NiCl_2$, and $ZnSO_4$ are not effective. Only $CoCl_2$ partially mimics the effect of $MnCl_2$[64]. $MnCl_2$ increases the production of GAP-43, peripherin[64], integrins[65], and the MAP kinases ERK1 and ERK2[66] on the differentiation of PC12 cells. Furthermore, $MnCl_2$ induces differentiation of PC12 cells harvested on laminin-coated culture plates, but not on poly-D-lysine–coated culture plates[64]. These studies suggest that the differentiation induced by $MnCl_2$ in PC12 cells is associated with interactions between the extracellular matrix and integrins[64]. However, association of $MnCl_2$-induced differentiation with hypoxia and ROS has not been reported. Co(II) and Mn(II) were shown to increase erythropoietin expression in the human hepatoma cell line Hep3B, but zinc, iron, cadmium, and tin did not increase erythropoietin expression to the degree that hypoxia did[16]. $MnCl_2$ also has been reported to induce up-regulation of glyceraldehyde-3-phosphate dehydrogenase (GAPDH), one of the hypoxia-induced genes[67]. ROS generated by cobalt chloride was reported to be associated with neurite outgrowth and up-regulation of ET-2/VIC, another hypoxia-induced gene[13], in PC12 cells[21].

Although $NiCl_2$ is known as a hypoxia mimetic agent, this divalent cation salt failed to induce neurite outgrowth in PC12 cells[64]. However, in that study, a 10-μ*M* concentration of $NiCl_2$ was used, whereas in other studies cobalt and manganese concentrations were 300 μ*M*[64]. The concentration of nickel needed to achieve a hypoxic effect was reported to be higher than that of cobalt or manganese[16]. Thus, Ni(II) at a concentration as low as 10 μ*M* might be insufficient to induce neurite outgrowth in PC12 cells.

Co(II) acts as a pro-oxidant, as does Fe(II, III) and Cu(II), and promotes lipid peroxidation. In general, these metals have been thought to produce cytotoxic activity through the stimulation of ROS generation and to induce cell death at high concentrations. In fact, $CoCl_2$ at 500 μ*M* previously has been reported to induce apoptosis in PC12 cells through ROS[54,68]. However, $CoCl_2$ also was reported to reduce the cytotoxic effect of epigallocatechin gallate or chlorogenic acid against cancer cells and to promote the growth of the cells, but $FeCl_3$ and $CuCl_2$ did not affect cytotoxicity[69,70]. The radical species derived from chlorogenic acid were transformed into new radical species or were completely scavenged by $CoCl_2$ at concentrations between 30 μ*M* and 30 m*M*[71]. These results suggest that new radical species generated by the reaction of $CoCl_2$ with chlorogenic acid or through the radical scavenging activity of $CoCl_2$ play a role in the inhibition of the cytotoxic effect of agents such as epigallocatechin gallate or chlorogenic acid against cancer cells. Furthermore, $CoCl_2$ at 50 m*M* reacts with antioxidants such as ascorbic acid and produces radicals at higher concentration than does 500 m*M* Fe(III). However, $NiCl_2$ at 50 to 100 m*M* does not produce ascorbic acid radicals[72]. Intracellular ROS induced by $NiCl_2$ also were reported to be produced at remarkably lower levels than those induced by $CoCl_2$[73]. Hence, it appears that the actions of cobalt differ from those of other metals in some cases. ROS participate in the induction not only of cell death, but also cell differentiation[21,51], as described above. Based on these reports, the amount or character of the ROS generated by nickel at a lower concentration and by other metals (except for manganese and cobalt) may be insufficient to induce differentiation in PC12 cells. Furthermore, organic radicals produced by the reaction of $CoCl_2$ with components of the culture medium may be involved in the neurite outgrowth of PC12 cells. Confirmation of these theories deserves future study.

## ACKNOWLEDGMENTS

K.S. and E. K.-N. gratefully thank Drs. Kazunori Nakamura, Hiroshi Kuriyama, Masahiro Iwakura, and Etsuo Niki of AIST for their support. E. K.-N. is a recipient of a New Energy and Industrial Technology Development Organization (NEDO) fellowship. This work was supported in part by a grant-in-aid for scientific research (Number 17580089) from the Japan Society for the Promotion of Science.

## BIOSKETCHES

**Eiichi Kotake-Nara, PhD,** is a NEDO postdoctoral fellow at the New Energy and Industrial Technology Development Organization (NEDO), 16F, MUZA KAWASAKI Central Tower, 1310 Omiya-cho, Saiwai-ku, Kawasaki, Kanagawa 212-8554, Japan and at the Institute for Biological Resources and Functions, National Institute of Advanced Industrial Science and Technology (AIST), Tsukuba Central 6, 1-1-1 Higashi, Ibaraki 305-8566, Japan. E-mail: eiichi-nara@aist.go.jp

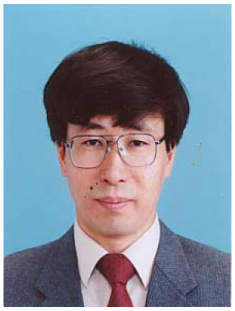

**Kaname Saida, PhD,** is a chief researcher at the Institute for Biological Resources and Functions, National Institute of Advanced Industrial Science and Technology (AIST), Tsukuba Central 6, 1-1-1 Higashi, Ibaraki 305-8566, Japan. E-mail: k.saida@aist.go.jp
